# Early identification of a ward-based outbreak of *Clostridioides difficile* using prospective multilocus sequence type-based Oxford Nanopore genomic surveillance

**DOI:** 10.1017/ice.2024.77

**Published:** 2024-09

**Authors:** Max Bloomfield, Samantha Hutton, Megan Burton, Claire Tarring, Charles Velasco, Carolyn Clissold, Michelle Balm, Matthew Kelly, Donia Macartney-Coxson, Rhys White

**Affiliations:** 1 Awanui Labs Wellington, Department of Microbiology and Molecular Pathology, Wellington, New Zealand; 2 Te Whatu Ora/Health New Zealand, Infection Prevention and Control, Capital, Coast and Hutt Valley, Wellington, New Zealand; 3 Institute of Environmental Science and Research, Health Group, Porirua, New Zealand

## Abstract

**Objective:**

To describe an outbreak of sequence type (ST)2 *Clostridioides difficile* infection (CDI) detected by a recently implemented multilocus sequence type (MLST)-based prospective genomic surveillance system using Oxford Nanopore Technologies (ONT) sequencing.

**Setting:**

Hemato-oncology ward of a public tertiary referral centre.

**Methods:**

From February 2022, we began prospectively sequencing all *C. difficile* isolated from inpatients at our institution on the ONT MinION device, with the output being an MLST. Bed-movement data are used to construct real-time ST-specific incidence charts based on ward exposures over the preceding three months.

**Results:**

Between February and October 2022, 76 of 118 (64.4%) CDI cases were successfully sequenced. There was wide ST variation across cases and the hospital, with only four different STs being seen in >4 patients. A clear predominance of ST2 CDI cases emerged among patients with exposure to our hemato-oncology ward between May and October 2022, which totalled ten patients. There was no detectable rise in overall CDI incidence for the ward or hospital due to the outbreak. Following a change in cleaning product to an accelerated hydrogen peroxide wipe and several other interventions, no further outbreak-associated ST2 cases were detected. A retrospective phylogenetic analysis using original sequence data showed clustering of the suspected outbreak cases, with the exception of two cases that were retrospectively excluded from the outbreak.

**Conclusions:**

Prospective genomic surveillance of *C. difficile* using ONT sequencing permitted the identification of an outbreak of ST2 CDI that would have otherwise gone undetected.

## Background

*Clostridioides difficile* infection (CDI) represents a significant threat to the safe delivery of modern healthcare.^1,2^ Most CDI is genomically diverse;^3^ however, a significant minority is associated with inpatient transmission events, and hospital outbreaks are well-described.^4,5^ *C. difficile* is difficult to eradicate from the environment and healthcare workers’ hands due to its propensity to form spores.^1,6,7^ Residence in a room following a patient with CDI is a risk factor for acquiring the infection,^8–10^ supporting the importance of inpatient transmission of *C. difficile*. Detecting outbreaks at an early stage based on incidence alone has performed poorly.^4,11,12^ Genomic sequencing of isolates offers the ability to detect transmission events at an earlier stage; however, genomic surveillance for *C. difficile* has predominantly been performed in central reference laboratories or used in a retrospective fashion after an outbreak has reached sufficient size to be suspected based on incidence alone.^5,13–15^

We established a simplified prospective genomic surveillance program in our laboratory using Oxford Nanopore Technologies (ONT) sequencing to detect hospital-associated transmission events to allow early remedial action from hospital infection prevention and control (IPC). This surveillance program targets several hospital-associated organisms, including *C. difficile,* in a weekly sequencing run, with the primary output being a multilocus sequence type (MLST).^16^ Currently, there are 1,169 unique *C. difficile* MLST profiles described on PubMLST.^17,18^ As described previously, this sequencing program was established at relatively low-cost and staffing requirement and without prior bioinformatics experience.^16^ Here, we describe an outbreak of *C. difficile* detected by this program, which enabled effective IPC intervention.

## Methods

### Setting

Wellington Regional Hospital (WRH) provides tertiary services to the lower north island of New Zealand (NZ), serving a population of around 500,000. Ward B provides inpatient care for patients admitted under the hematology, oncology, and renal services. Both allogeneic hemopoietic stem cell and kidney transplantation are performed. Ward B has 39 beds arranged between three separate areas, with 12 single rooms, 10 of which have their own toilet. The remaining rooms are double or four-bedded rooms, where the single toilet is shared with up to three others. Nursing staff do not move between areas, but medical and allied health (e.g. physiotherapy) staff do. Standard and Transmission-Based Precautions^19^ were in use prior to the outbreak, and patients with diarrhea were managed with Contact Precautions (CP). Patients in CP had daily bathroom cleaning with a dilute chlorine formula (Divercleanse, Diversey, Fort Mill, SC, United States), and other general room, equipment, and ward cleaning was with a non-chlorine cleaner (Wipeout, Diversey) and detergent wipes (Neutral Detergent Wipes, Reynard, Havelock North, NZ). Gloves and gowns/aprons were used if there was risk of blood or body fluid exposure but not mandated at other times for patients in CP.

Awanui Laboratories Wellington is a medium-sized laboratory, which provides clinical diagnostic services to WRH and the local region. The microbiology and molecular departments process around 300,000 samples yearly.

### Genomic surveillance program

From February 2022 we began prospectively sequencing all *C. difficile* isolated from inpatients at WRH. Our laboratory performs reflexive *C. difficile* testing (*C. DIFF QUIK CHEK COMPLETE*®, TechLab, Blacksburg, VA, United States) on all diarrheal stool samples from hospital patients. Our method has been described previously;^16^ briefly, this involves culture from stool on chromID^TM^ *C. difficile* agar (bioMerieux, Marcy-l’Etoile, France) from patients with a positive glutamate dehydrogenase antigen, with subsequent DNA extraction, library preparation and sequencing on the ONT MinION device (further detail in Supplementary Materials). An MLST is generated from the raw reads using Krocus v1.0.3.^20^ After each sequencing run MLST data are matched to patient bed movement data obtained from our hospital electronic data warehouse, which captures the time of all inpatient bed movements. Any wards that cases have spent >24 hours on in the last three months are recorded. This allows real-time monitoring of the incidence of specific *C. difficile* STs according to patients’ recent ward exposures. If greater than expected numbers of a given *C. difficile* ST are observed related to a given ward, then further investigations are instigated by the IPC team.

### IPC changes implemented

In response to the increase in ST2 CDI cases, several changes to IPC practices were implemented on Ward B. Staff were reminded to place patients with diarrhea in CP in single rooms with a dedicated toilet. Hand hygiene messaging and auditing were increased, and the routine use of gloves and aprons/gowns for all room entries was mandated for patients with diarrhea. The general ward and equipment cleaning product was changed to an accelerated hydrogen peroxide (aHP) wipe (Oxivir® Tb wipes, Diversey). Discharge cleaning protocols and the established fluorescent marker cleaning audits were reviewed and regularly monitored with the cleaning contractor. Shared patient equipment cleaning was reviewed to ensure that all shared items were allocated to appropriate ward personnel for cleaning. These changes were initiated in August 2022, except for the cleaning product change which occurred in October 2022 due to global product shortages of the aHP wipes.

### Retrospective phylogenetic analysis

In late 2023 further genomic analysis was undertaken using the original sequence data to compare the outbreak cluster to the wider phylogeny of *C. difficile* within our institution. Original Fast5 sequence files were converted to Pod5 using pod5 v0.3.2 (https://github.com/nanoporetech/pod5-file-format, accessed 09 January 2024) and then basecalled using Dorado v0.4.3 (https://github.com/nanoporetech/dorado, accessed on 08 January 2024) with the “super-accuracy” model. The original DNA extracts for seven outbreak isolates were still available, so were resequenced (R10.4.1 flow cell, kit SQK-RBK114.96) to provide additional depth of coverage. Pod5s were basecalled in the same way as above and sequence data combined. Further details of laboratory methods including read quality control, genome assembly, multilocus sequence typing, virulence and antibiotic resistance gene genotyping, genome annotation, public data curation, and phylogenetic analysis are available in the Supplementary Materials. Briefly, phylogenetic trees were built from a core-genome alignment (Parsnp v1.7.421 or SPANDx v4.022), and constructed with RAxML (GTR-GAMMA model; v8.2.1223). The analysis and reporting of this outbreak constituted an “audit or related activity” as per NZ Health and Disability Ethics Committees, so did not require review.

## Results

**Figure 1.**
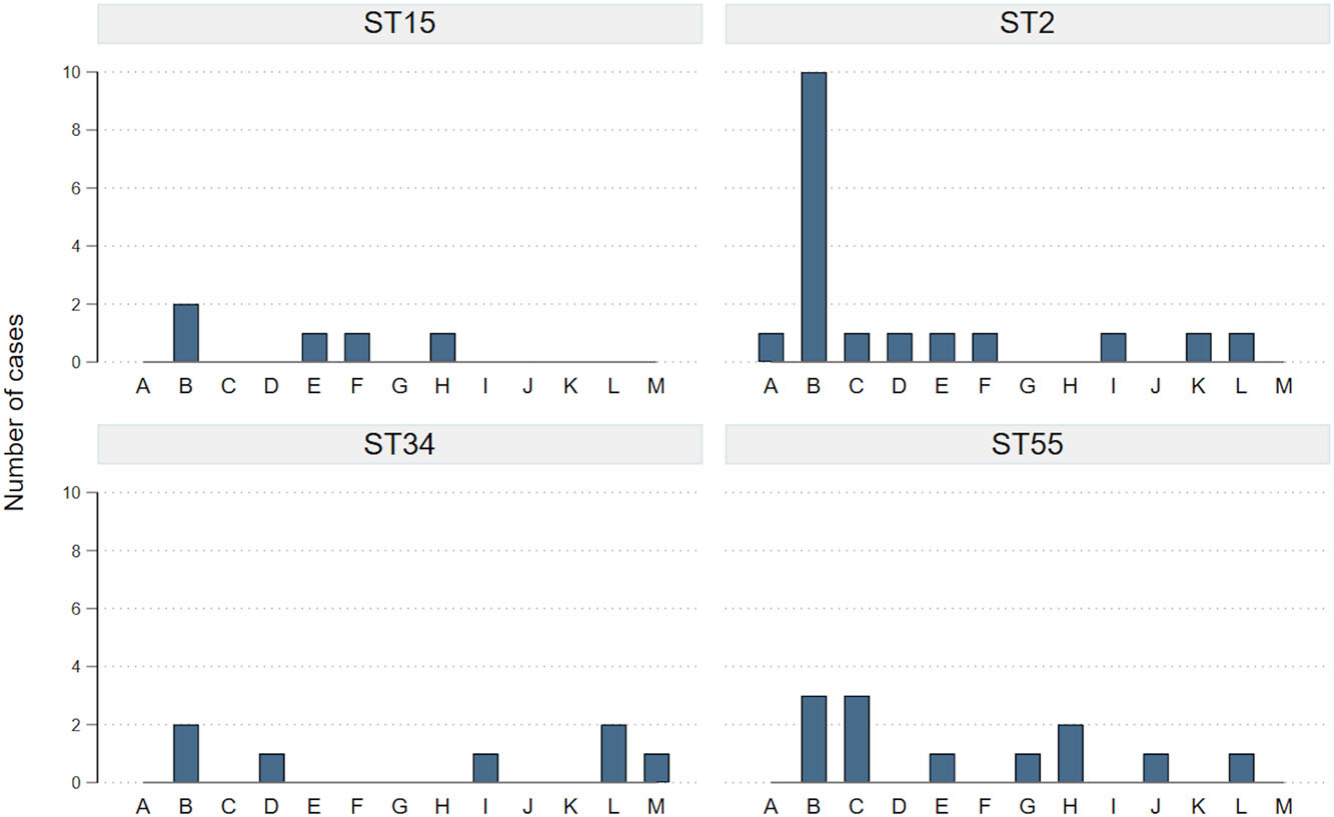
Ward exposures for the four commonest sequence types over the three-month period prior to *Clostridioides difficile* positive sample. Cases may have exposure to multiple wards, hence total number of cases per ST in the figure equals more than the total number of individual cases. Individual wards displayed on the x axis. ST, sequence type.

From February to October 2022 89 isolates of *C. difficile* from WRH inpatients were available for sequencing (Supplementary Materials, Table S1), which represented 75.4% of all cases (Table 1). Only four STs were seen in >4 patients, with ST2 being the commonest (14 cases, 18.4%). No ST1 *C. difficile* (associated with ribotype 027) was detected. Figure 1 shows recent ward exposures for those with common STs. Of these, there was a relatively even distribution of ward exposures across STs, except for ST2, for which exposure to Ward B was much more common. As this association emerged, patient bed movements were examined by the IPC team in detail, revealing multiple occasions where patients were present in the same area of Ward B at the same time (Supplementary Materials, Figure S1).

**Table 1.** Multi-Locus Sequence Typing Results for *Clostridioides difficile* at Wellington Regional Hospital from February to October 2022

	*N*	%
Total *C. difficile* infection cases	118	
*C. difficile* recovered in culture	89	75.4
Multilocus sequence type achieved	76	64.4
Sequence types seen in >4 patients		
2	14	18.4^a^
55	6	7.9
34	6	7.9
15	5	6.6
Other	45	59.2

a Percentage is of those where MLST achieved.

**Figure 2.**
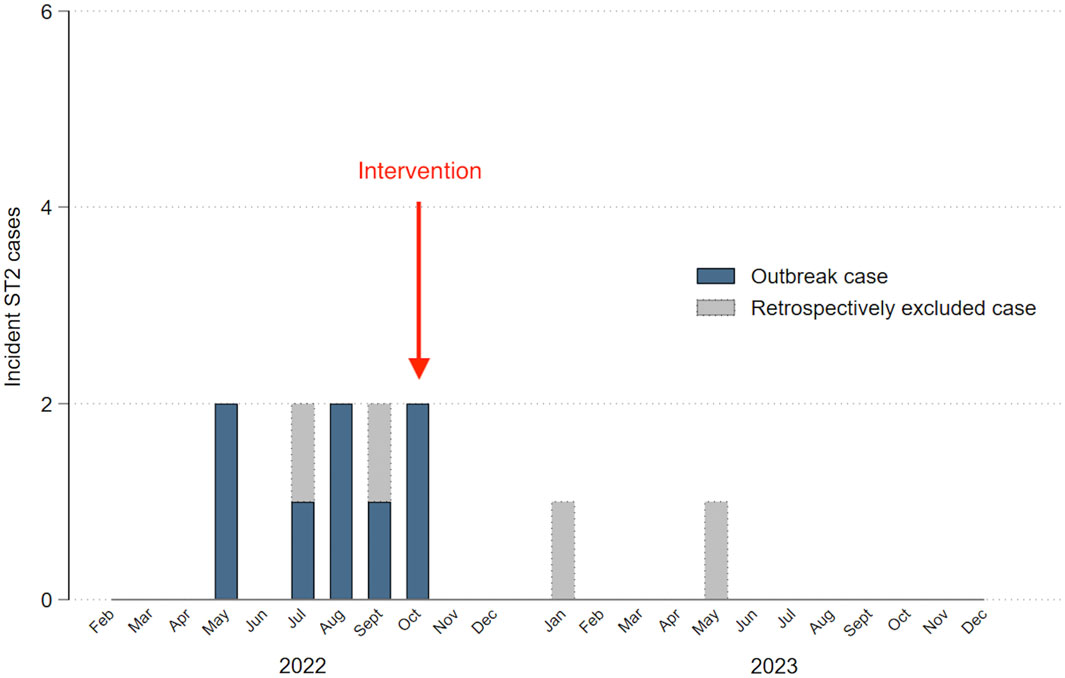
Incidence of sequence type (ST)2 *Clostridioides difficile* infection in patients with exposure to Ward B in the three months prior to diagnosis. “Intervention” denotes when all of the changes to infection control practices on Ward B had been implemented. “Retrospectively excluded case” applies to ST2 cases that were initially thought to be part of the outbreak, but were excluded in the retrospective phylogenetic analysis.

In August 2022, when there were six known Ward B-associated ST2 cases, the IPC team decided to intervene. Due to supply problems, the change in cleaning product and other measures were not fully implemented until October 2022. Incident cases of ST2 CDI with exposure to Ward B from February 2022 until December 2023 are shown in Figure 2. Following the intervention, the incidence of ST2 CDI on Ward B rapidly declined.

**Figure 3.**
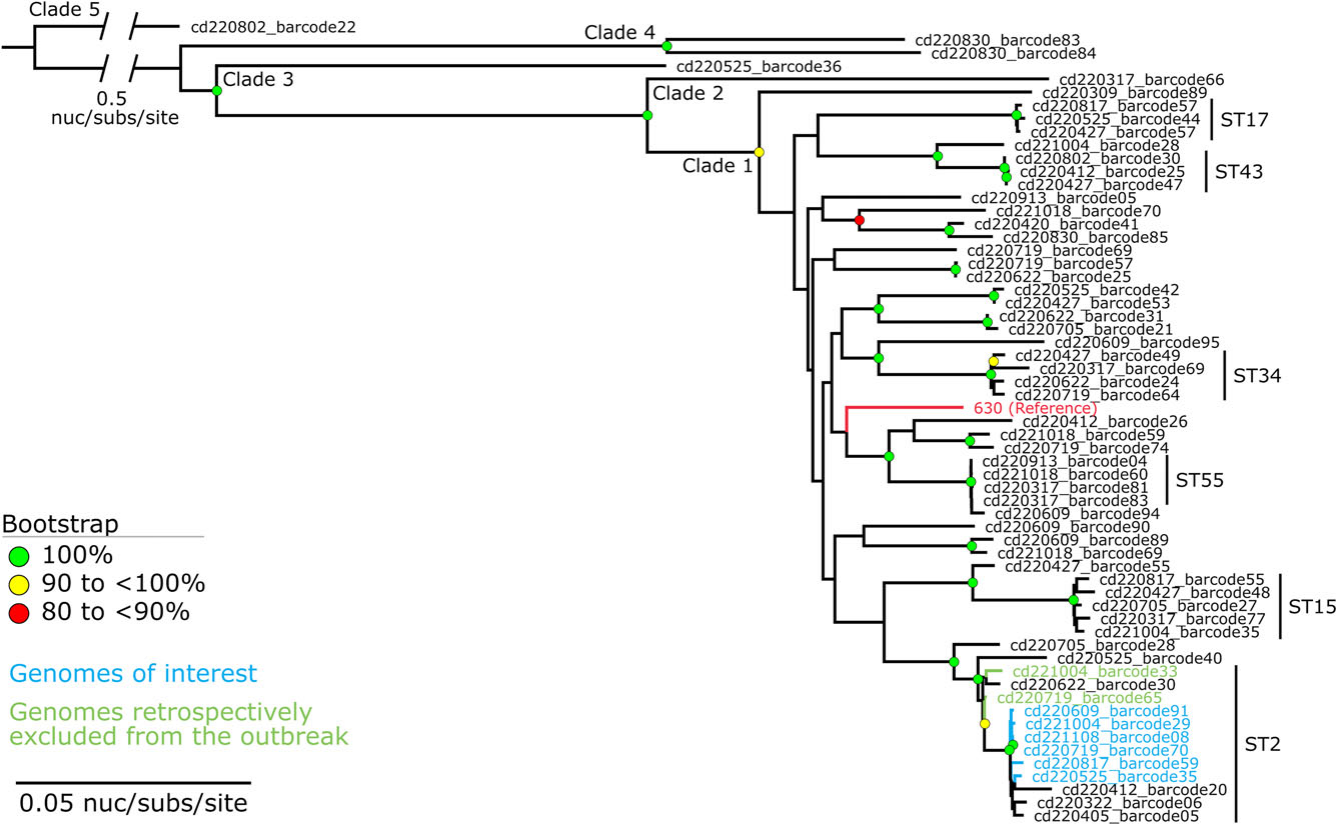
Maximum likelihood phylogeny of *Clostridioides difficile* cases identified in Wellington Regional Hospital. The phylogeny was inferred from 37,441 core-genome single-nucleotide variants (SNVs) from 61 genomes. SNVs were derived from a core-genome alignment of 954,405 bp and are called against the chromosome of strain 630 (GenBank: AM180355). Phylogeny was rooted according to the actual root by *C. difficile* strain cd220802_barcode22 (SRA: SRR27352627), which has been truncated for visualization. Bootstrap values >80% (1,000 replicates) are shown. Isolates in blue represent the ST2 cases of interest on Ward B. Isolates in green represent those retrospectively excluded from the outbreak due to forming outgroups from the main outbreak cluster. ST2 isolates found subsequent to the infection control interventions are omitted from this figure.

For the retrospective phylogenetic analysis, of the 89 sequenced isolates, 20 genomes were excluded due to estimated sequence coverage of <10×, and a further nine were omitted as the total genome length fell outside the first quartile and third quartile in combination with 1.5× the interquartile range (i.e., <3,841,755 bp and >4,525,475 bp) (Supplementary Materials, Table S2), as derived from the genome lengths of the 69 *C. difficile* genomes (Supplementary Materials, Table S3). An alignment of 37,441 core-genome single-nucleotide variants (SNVs) was used to construct a maximum likelihood phylogenetic tree from the remaining 60 genomes, which included eight of the original ten outbreak isolates (Figure 3). This revealed that two ST2 cases did not cluster with the other outbreak isolates, so are likely to represent co-incidental sporadic ST2 cases on Ward B during the outbreak time period. The two ST2 cases detected on Ward B subsequent to the IPC intervention also did not cluster with the outbreak isolates (Figure 2).

Given the absence of an available ST2 reference genome representing NZ *C. difficile* strains, we selected cd231108_barcode08 (GenBank: CP144679) as our reference genome (endpoint case on Ward B identified on 24th October 2022). Detailed genomic information, including sequencing results, antibiotic resistance gene analysis, and genome features such as a circular chromosome (Supplementary Materials, Figure S2), prophage, and plasmid, can be found in the supplementary materials (Supplementary Results). With the availability of the cd221108_barcode08 reference genome, we aimed to contextualize our ST2 genomes from WRH on a global scale (Supplementary Materials, Figure S3). Most ST2 genomes align with L1 lineage (definitions according to Xu et al.^24^). Notably, cd221004_barcode33 falls into L2, specifically SL2b. Furthermore, seven WRH genomes form a distinct subclade of interest, warranting further investigation (Supplementary Materials, Figure S4). Analysis of high-quality publicly available Illumina sequence data suggests that the primary ST2 cluster on Ward B (cd220322_barcode06, cd220405_barcode05, cd220525_barcode35, cd220817_barcode59) likely shares a common ancestor with ST2 genomes from Australia.

**Figure 4.**
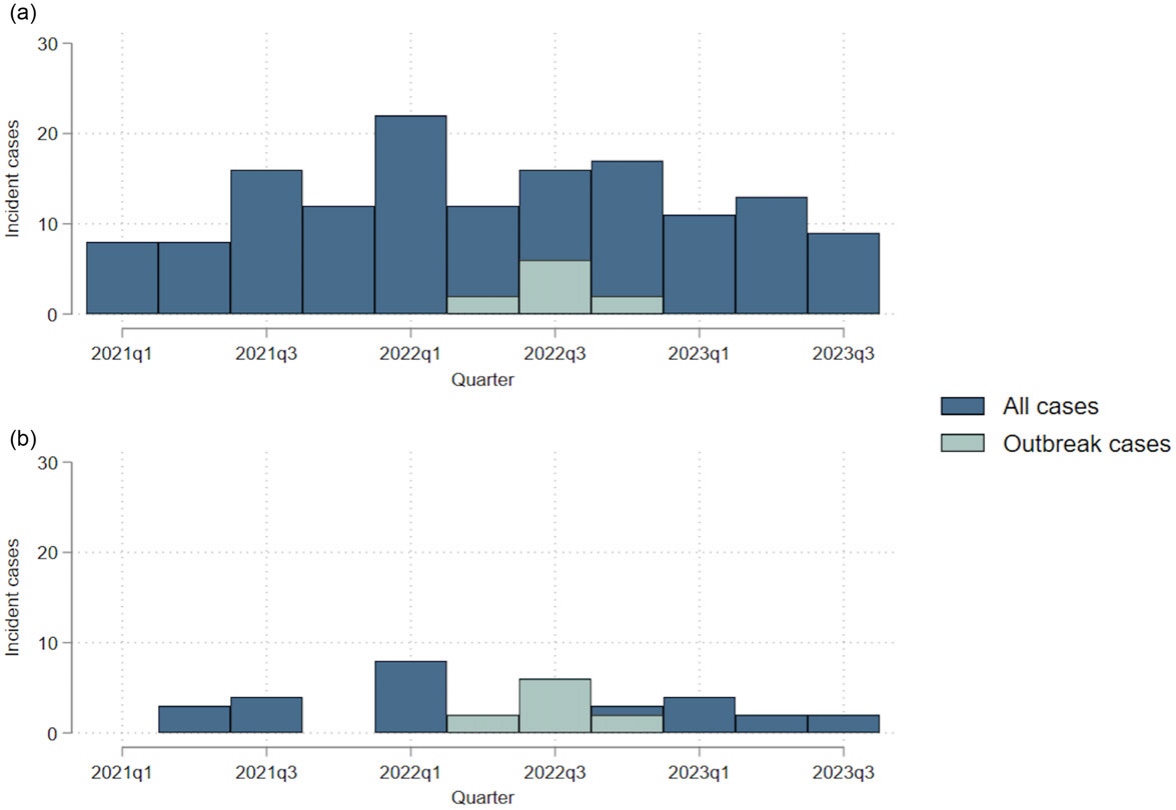
Incidence by quarter of outbreak cases compared to all CDI cases within a) the whole hospital, b) Ward B.

Figure 4 shows the ST2 cases associated with this outbreak compared to total incident CDI within the wider hospital and on Ward B. The outbreak cases did not generate an obvious rise in incident cases above baseline variability seen in incidence in the hospital or on Ward B.

## Discussion

This report has demonstrated the detection of a sustained outbreak of *C. difficile* ST2 over a six-month period on a single ward using prospective MLST-based surveillance. This outbreak did not generate a clear rise above baseline variability in CDI incidence, so would almost certainly have gone unnoticed (or increased in size to become detectable) if surveillance was based on overall CDI incidence alone. The use of early warning algorithms based on incidence and epidemiological data have been proposed, however have been shown to perform poorly for detecting in-hospital transmission events of *C. difficile.* ^4,11,12^ In our approach we were able to combine basic low-discrimination genomic analysis with high-quality epidemiological data to detect the outbreak and initiate remedial IPC actions when there were only six suspected cases, and the outbreak was halted when it had reached eight cases.

The use of genomics is well-described for investigating hospital outbreaks of *C. difficile*, however most reported investigations were performed retrospectively, either over a defined time period, or instituted once an outbreak had reached sufficient size to generate a clear rise in CDI incidence.^4,5,15,25,26^ Prospective PCR-ribotype-based or whole-genome sequencing surveillance is commonly performed at the reference laboratory level, however this is typically intended to monitor the wider genomic epidemiology of *C. difficile*, rather than to detect individual hospital outbreaks.^13,14^ Here we describe the potential value of a prospective genomic surveillance system, which can be implemented at the level of a medium-sized front-line clinical microbiology laboratory. This system was implemented at relatively low-cost and additional staffing requirement and without the need for specialist bioinformaticians to analyze data.^16^ Furthermore, we are not aware of prior reports where surveillance has been performed using solely ONT sequencing, which represents a potentially more accessible sequencing option for smaller hospital laboratories such as ours, due to low capital costs and laboratory space requirements. However, it should be noted that the requirement for a cultured isolate could represent a barrier for other laboratories wishing to adopt genomic surveillance for *C. difficile*. Most clinical laboratories use culture-independent methods for CDI diagnosis,^27^ meaning culture is not part of the standard diagnostic pathway. Although our laboratory had experience with *C. difficile* culture prior to implementing genomic surveillance, we believe the approach of reflex culture on antigen- or PCR-positive stool samples would be relatively straightforward to implement from a workflow perspective for many diagnostic laboratories. We employ a simple 48-hour direct-plating culture method, without other more complicated steps such as alcohol/heat shock,^16^ and the anaerobic environment is created using disposable gas packs and small containers, which requires minimal capital expenditure compared to a dedicated anaerobic incubator.

A limitation of our approach is the relatively nondiscriminatory nature of MLST, which cannot be used to infer individual patient transmission events.^28^ This was demonstrated in the retrospective phylogenetic analysis, where two of the ST2 cases did not cluster with the other isolates. However, when combined with epidemiological data, the additional level of detail afforded by MLST allowed increases in incidence at the ST-level to be appreciated at a stage where increases in incidence at the organism-level were inapparent. Our data showed a high degree of genomic/ST variability within *C. difficile* detected in our hospital, which is consistent with prior reports.^3^ This is why we were able to detect this outbreak at a relatively early stage using an MLST-based approach; there was a clear change at the ward-level from the expected variability in the STs detected. In jurisdictions where hospital CDI is dominated by few STs (if these exist), or where electronic capture of bed movements is not available, an MLST approach like this may be less effective.

ONT sequencing technology is constantly developing. Most sequence data for this investigation was generated using older chemistry and flow cells (R9.4.1) than are currently available. While a lower raw-read accuracy was achieved than with other platforms such as Illumina,^29^ it was sufficient to show clustering of the isolates within the wider hospital phylogeny (Figure 3). However, we were not able to generate precise SNV distances. ONT accuracy has now been shown to perform well for higher-resolution typing, such as core-genome MLST using the newer Q20+ chemistry and duplex basecalling.^30^ Since the outbreak reported here, we have moved our surveillance program to Q20+ chemistry and are planning to move to a higher-resolution typing method, which we anticipate will enable outbreaks or patient-to-patient transmission events to be detected even earlier, and will enable early exclusion of unrelated cases with the same MLST.

Around one-third of CDI cases could not be typed, largely due to negative culture (Table 1). This is similar to another sequencing study using the same culture media, where 62% of cases could be typed.^4^ This highlights a difficulty intrinsic to *C. difficile* genomic surveillance, and as a result, additional outbreak cases or transmission chains may have gone undetected. Furthermore, our surveillance did not test asymptomatic patients for *C. difficile* carriage, who could also contribute to transmission.^31^ These limitations may have delayed recognition of the outbreak or other transmission chains on the ward or within the hospital. Prior studies examining direct plating culture methods using chromID^TM^ *C. difficile* agar have found recovery rates above 90%, so our culture recovery rate of 75.4% can likely be improved on.^32,33^

The early detection of this outbreak allowed our IPC team to implement a range of interventions which was followed by a decrease in ST2-associated cases. Our institution has low rates of CDI by international comparisons, however this outbreak occurred on our hemato-oncology ward, where vulnerable patients, broad-spectrum antibiotic use, diarrhea, and long/repeated admissions are common.^34^ CDI poses unique challenges in IPC. Asymptomatic colonization is common, and antimicrobial exposure can increase environmental shedding of *C. difficile* spores.^31,35^ Patients who develop CDI will shed spores for several weeks after their diarrhea resolves and spores can survive in the environment for months-years.^36,37^ Transmission has been shown to be higher in patients where prior room occupants had CDI despite room cleaning at discharge.^10^ Additionally, CDI risk is also higher where the prior room occupant had broad-spectrum antibiotics independent of CDI status,^38,39^ highlighting the contribution to environmental contamination from asymptomatically colonized patients. In this outbreak transmission events were not clearly linked to particular rooms, which suggested more widespread ward contamination and that transmission from staff or shared equipment were contributing factors.

Chemical disinfection with sporicidal agents is effective in controlling CDI in endemic and outbreak settings.^1^ In an evaluation of four products, in both wipe and spray format, aHP showed the greatest reduction in viable spores and cleaning efficiency on surfaces for endemic and hypervirulent *C. difficile* strains.^40^ Implementation of aHP wipes for routine and equipment cleaning in addition to preexisting hypochlorite-based terminal cleaning was likely an important intervention in our setting. The aHP wipes were reported to be convenient and easy to use by staff performing cleaning tasks, particularly as they enabled a one-step process and were nontoxic to use. Similarly, the use of gloves for all room entries for patients with diarrhea (irrespective of cause) may have reduced *C. difficile* transmission more than would occur with improved hand hygiene alone.^41,42^ Given there were multiple components to the intervention it is not possible to determine which were more or less effective in this specific context. Furthermore, due to the low number of cases involved, we also cannot exclude a natural decline due to stochastic effects, however the sharp change in incidence after intervention supports practice improvements as the cause.

In this study, we have demonstrated the potential value of simplified prospective genomic surveillance for CDI using ONT sequencing, which detected an outbreak of CDI on a high-risk ward, which would have otherwise gone undetected. Whether these results can be applied to other settings and whether surveillance may be beneficial in lower-risk settings than a hemato-oncology ward remains to be determined, however, the additional level of detail afforded by sequencing allowed recognition of the outbreak, which in turn permitted effective intervention.

## Supporting information

Bloomfield et al. supplementary material 1 Bloomfield et al. supplementary material

Bloomfield et al. supplementary material 2 Bloomfield et al. supplementary material

Bloomfield et al. supplementary material 3 Bloomfield et al. supplementary material

Bloomfield et al. supplementary material 4 Bloomfield et al. supplementary material

Bloomfield et al. supplementary material 5 Bloomfield et al. supplementary material

## Data Availability

The study sequences are available in the NCBI under BioProject accession number PRJNA1057299. Raw Illumina sequence read data generated in this study have been deposited to the NCBI SRA under the accession numbers SRR27352554 to SRR27352669.

## Supplementary material

To view supplementary material for this article, please visit https://doi.org/10.1017/ice.2024.77
