# Supplementary material for: Early identification of a ward-based outbreak of Clostridioides difficile using prospective multilocus sequence type-based Oxford Nanopore genomic surveillance
Source: Infect Control Hosp Epidemiol. 2024 May 6;45(9):1057–63. doi: 10.1017/ice.2024.77 (PMC11518675; doi:10.1017/ice.2024.77)
Supplement: Bloomfield et al. supplementary material 1 — Bloomfield et al. supplementary material [file S0899823X24000771sup001.pdf]

Supplementary appendix

A ward-based outbreak of ST2 *Clostridioides difficile* identified and controlled via prospective MLST-based genomic surveillance using Oxford Nanopore sequencing

Author names

Max Bloomfield[1,2], Samantha Hutton[1], Megan Burton[1], Claire Tarring[1], Charles Velasco[1], Carolyn Clissold[2], Michelle Balm[1,2], Matthew Kelly[2], Donia Macartney-Coxson[3], Rhys White[3]

Affiliation

[1]Awanui Labs Wellington, Department of Microbiology and Molecular Pathology, Wellington 6021, New Zealand

[2]Te Whatu Ora/Health New Zealand, Infection Prevention and Control, Capital, Coast & Hutt Valley, Wellington 6021, New Zealand

[3]Institute of Environmental Science and Research, Health Group, Porirua 5022, New Zealand

Corresponding author

*Corresponding author: Max Bloomfield, Awanui Labs Wellington, Department of Microbiology and Molecular Pathology, Wellington 6021, New Zealand; Telephone: +64-4-381-5900; E-mail: maxim.bloomfield@ccdhb.org.nz

<u>This file includes the following:</u>

Supplementary Methods.

Supplementary Results.

Supplementary Figure S1. Ward area locations of Ward B ST2 *Clostridioides difficile* infection cases over time.

Supplementary Figure S2. Major genomic elements in *Clostridioides difficile* strain cd231108_barcode08.

Supplementary Figure S3. Maximum likelihood phylogeny of global *Clostridioides difficile* sequence type (ST)2 genomes.

Supplementary Figure S4. Maximum likelihood phylogeny of *Clostridioides difficile* sequence type (ST)2 cases identified in Wellington Regional Hospital and placed into global context.

Supplementary Methods

DNA extraction, library preparation and sequencing

Genomic DNA extraction involves suspending a 10µL loop of bacteria in 300uL of phosphate-buffered saline (PBS) and freezing at -20°C for at least 24 hours. The thawed solution is vortexed for one minute with a small quantity of 0.1mm zirconia/silica beads (dnature, Gisborne, New Zealand; SKU 11079101Z) and then centrifuged at 12,000 g for 30 seconds, with the supernatant stored at 4°C for sequencing without further clean-up.

Library preparation occurs immediately prior to the weekly sequencing run. Qubit gDNA quantification of stored extracts is performed using the dsDNA BR kit, with subsequent normalization. Preparation, flow cell loading, and sequencing are per the manufacturer's instructions, using the Rapid Barcoding Kit 96 (SQK-RBK110.96) and MinION flow cell (FLO-MIN106D). The run is stopped when sufficient sequence is available.

Quality control and trimming of the nanopore sequence read data

Raw nanopore reads were checked for quality using NanoPlot v1.40.0[1]. Initial trimming was performed using NanoFilt 2.8.0[1], where 52 nucleotides were removed from the start and end of each read to remove adapters and barcodes. Subsequently, Filtlong v0.2.1 (https://github.com/rrwick/Filtlong, accessed 08 January 2024) was utilised to discard the lowest 5% of reads (--keep_percent 95), retaining the top 95% based on read-base quality assessment. Any remaining reads that were less than 200 base pairs in length were also removed. NanoStat v1.6.0[1] was used to perform a quality assessment on the trimmed and filtered nanopore reads. To perform taxonomic profiling and identify *C. difficile* by classifying DNA sequences, we used Kraken v2.1.2[2] with default parameters and an National Center for Biotechnology Information (NCBI) Reference Sequence (RefSeq) database[3], PlusPFP (https://benlangmead.github.io/aws-indexes/k2, accessed on 01 December 2023). The database contained references for archaea, bacteria, human, viruses, plasmids, protozoa, fungi, plant, and the 'UniVec core' subset of the UniVec database (a database of vector, adaptor, linker and primer sequences). The sequence read data quality metrics for all 89 samples are presented in the supplementary materials (Supplementary Materials, Table S4).

Genome assembly

The trimmed and filtered reads were *de novo* assembled using Flye v2.9.2[4,5] with parameters set to: (i) estimate the genome size to 4.1 Mbp; and (ii) three polishing iterations. The assemblies underwent three rounds of additional polishing by mapping the corresponding nanopore reads to each contig using minimap2 v2.24[6,7], and then correcting single-nucleotide variations (SNVs) and insertions and deletions (INDELs) with racon v1.4.3[8]. A final round of polishing was completed using medaka v1.8.0 (https://github.com/nanoporetech/medaka, accessed on 01 December 2023). Assembly metrics were

assessed using QUAST v5.0.2[9]. The *de novo* assembly quality metrics are presented in the supplementary materials (Supplementary Materials, Table S3).

In silico genotyping

MLST v2.22.0 (https://github.com/tseemann/mlst, accessed on 09 January 2024) with default settings was used to characterise the MLST for each strain by querying the assemblies against the *C. difficile* MLST allelic profiles hosted on PubMLST[10,11]. ABRicate v1.0.1 (https://github.com/tseemann/abricate, accessed on 09 January 2024) was used to identify virulence (coverage/identity, ≥60%/≥90%), and acquired antibiotic resistance genes using (coverage/identity, ≥60%/≥90%), using the Virulence Factor Database[12] and ARG-ANNOT database[13], respectively (last updated 15 September 2023). Antibiotic resistance genes were also detected using the BacAnt v3.3.3 tool and the BacAnt-database v3.1[14].

Genome annotation for cd231108_barcode08

The assembly representing strain cd231108_barcode08 was annotated using Prokka v1.14.6[15], with the complete genome of *C. difficile* ST54 strain 630 (GenBank: AM180355) as a reference for trusted proteins. Prophage regions were identified using PHASTER[16,17] and then annotated using Pharokka v1.5.1[18]. Mobile Genetic Elements were identified using IslandViewer 4[19] and ISsaga v2.0[20] (ISfinder platform[21]); followed by manual curation using Artemis v18.2.0[22].

Assembly based phylogenetic analyses

For the *C. difficile* detected in our hospital, a core-genome alignment was generated using Parsnp v1.7.4[23], using the chromosome of 630 (GenBank: AM180355) as the reference to call SNVs. The resulting SNV alignment was used to reconstruct the phylogeny. A maximum likelihood phylogenetic tree was reconstructed using RaxML v8.2.12[24] (GTR-GAMMA correction) by optimising 20 distinct, randomised maximum parsimony trees before adding 1,000 bootstrap replicates. The resulting phylogenetic tree was visualised using FigTree v1.4.4 (http://tree.bio.ed.ac.uk/software/figtree/, accessed on 09 January 2024).

In addition to the 12 *C. difficile* ST2 genomes sequenced in this study, 10 complete ST2 genome assemblies were downloaded from the National Center for Biotechnology Information (NCBI) Assembly database. Another 2,579 publicly available *C. difficile* ST2 genomes (sequence data) were downloaded from the NCBI sequence read archive (SRA (https://www.ncbi.nlm.nih.gov/sra, accessed 09 January 2024)). Raw sequence reads were *de novo* assembled using Shovill v1.1.0 (https://github.com/tseemann/shovill, accessed on 09 January 2024), which utilises: Seqtk v1.3-r106 (https://github.com/lh3/seqtk, accessed on 09 January 2024); Trimmomatic v0.36[25]; Lighter v1.1.2[26];

FLASH v1.2.11[27]; SKESA v2.4.0[28,29]; Samclip v0.4.0 (https://github.com/tseemann/samclip, accessed 09 January 2024); SAMtools v1.16.1[30], Burrows-Wheeler Aligner (BWA) v0.7.17[31]; and Pilon v1.24[32].

Parsnp was used to generate a 2,166,128 bp core-genome alignment from 2,601 genomes. These genomes represent 12 ST2 genomes from WRH, 5,579 draft ST2 genomes, and 10 complete ST2 genomes. The resulting SNV alignment was used to reconstruct a maximum likelihood phylogenetic tree following the protocol outlined in the species-level phylogenetic analyses.

High-resolution cluster phylogenetic analyses

High-resolution analyses of genetic variants were performed using BWA; BEDTools v2.28.0[33]; seqtk; Trimmomatic; pindel[34]; Mosdepth[35]; SAMtools v1.9; Picard v2.7.1 (https://github.com/broadinstitute/picard, accessed on 10 March 2023); the Genome Analysis Tool Kit v4.3.0.0 (GATK)[36,37]; and SNPEff v4.3.1t[38], as implemented in SPANDx v4.0[39]. Notably, SNVs identified in highly dense SNV and/or predicted recombination regions were removed (i.e., genomic islands (n = 70 SNVs); prophage elements (n = 66 SNVs); conjugative transposons (n = 46 SNVs); cell wall protein (cwp) gene cluster (n = 28 SNVs); the pathogenicity locus (PaLoc) (n = 13 SNVs); Cd*ISt*1 genetic elements (n = 9 SNVs); *sigK* intervening (skin) element (n = 7 SNVs); and putative mobile elements (n = 2 SNVs)). Resulting SNV alignments were used to reconstruct phylogenies. A maximum likelihood phylogenetic tree was reconstructed using RaxML v8.2.12 (GTR-GAMMA correction) by optimising 20 distinct, randomised maximum parsimony trees before adding 1,000 bootstrap replicates. The resulting phylogenetic trees were visualised using FigTree v1.4.4. Notably, this analysis defines a core genome as regions estimated to the nearest 100 bp with ≥95% coverage across one or more genomes in the given population. The genome for cd220412_barcode20 (with an average coverage of 13x) was excluded to adopt a more conservative approach in the read-mapping SNV calling process.

Supplementary Results

A reference genome was generated for a *C. difficile* ST2 isolate

Following quality trimming and filtering, the nanopore sequencing produced 175,821 single-ended reads, ensuring complete genome coverage at an average depth of 220x. The median read length stood at 5,138 bp (N50 = 7,126 bp), and the median read quality score was 17.1. The *de novo* nanopore assembly revealed that cd231108_barcode08 possesses a circular chromosome with a length of 4,168,836 bp and an average GC content of 28.87% (Supplementary Materials, Figure S2). Furthermore, *in silico* analysis for antibiotic resistance genes detected a *vanG*-like cluster positioned at genome coordinates 1,770,450 to 1,776,603, along with a $bla_{\mathrm{CDD\text{-}1}}$ gene (encoding a beta-lactamase) found between genome coordinates 483,638 and 484,591. Additionally, whole-genome sequencing highlighted the presence of several significant elements: a major flagellar operon, a *sigK* intervening (skin) element, two genomic islands, and a 19,615 bp PaLoc carrying the *tcdA* and *tcdB* genes (encoding toxins). Moreover, the chromosome of cd231108_barcode08 contains a prophage (designated prophage 1), which is also found in the publicly available genomes Cd16 (GenBank: CP037812) and ASS22 (GenBank: CP133826 (Supplementary Materials, Figure S2). Notably, there are three conjugative transposons carried by the chromosome of cd231108_barcode08, which are conserved in the four other publicly available ST2 genomes (CDI-30 (GenBank: CP126067), Cd16, ASS22, and 08ACD0030 (GenBank: CP010888)). The complete genome of cd231108_barcode08 contains a 4,423 bp plasmid named pcd231108_barcode08A.

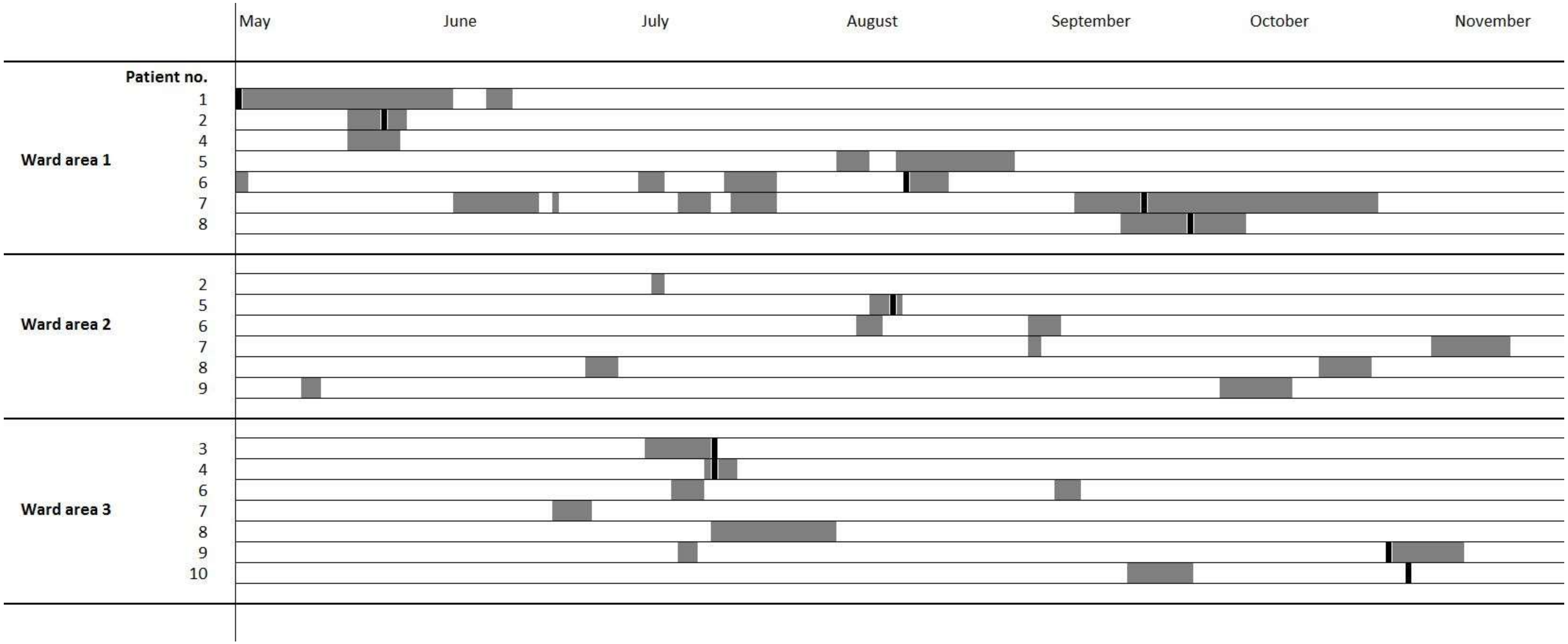

Supplementary Figure S1. Ward area locations of Ward B ST2 *Clostridioides difficile* infection cases over time. Solid black bars indicate the date of positive sample for *C. difficile* infection.

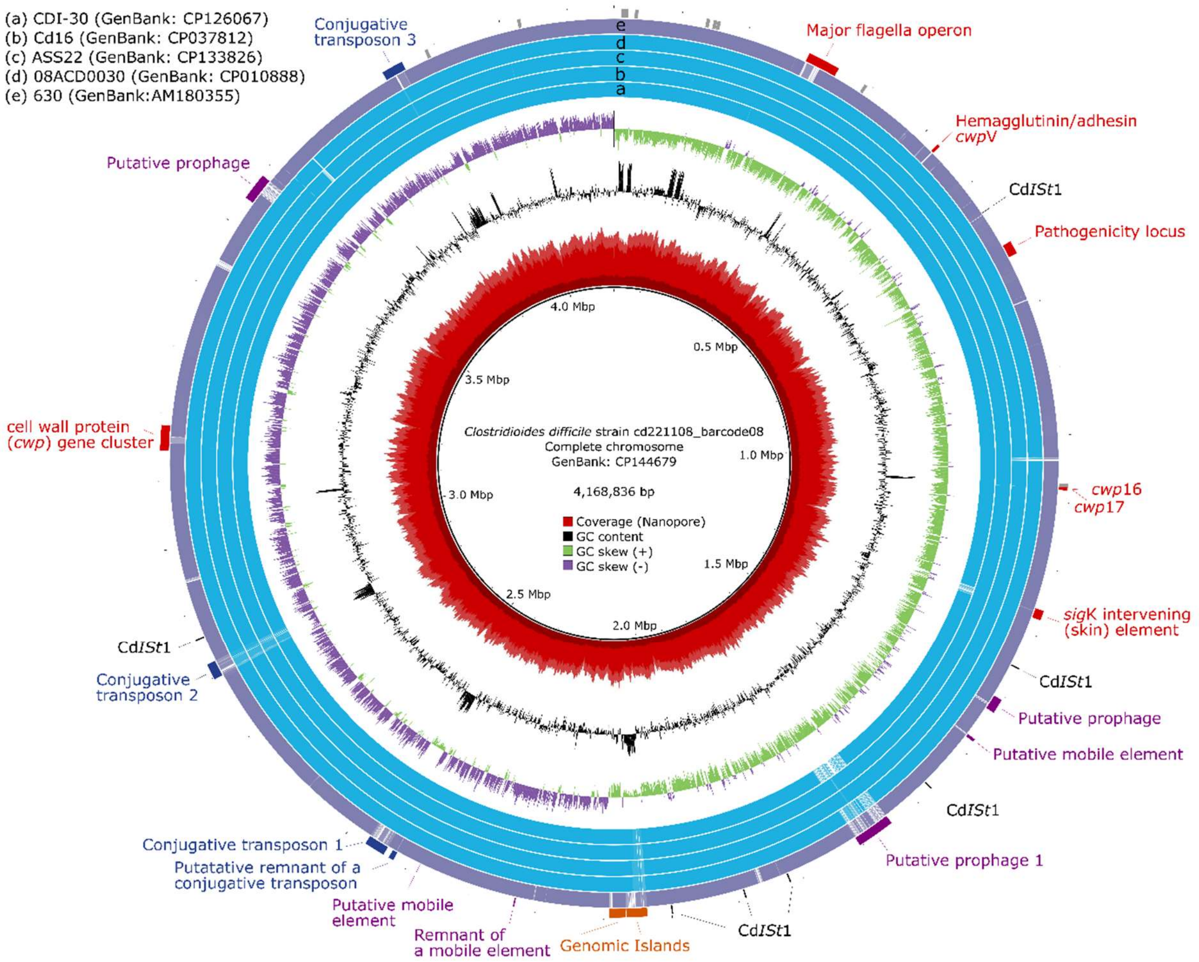

Supplementary Figure S2. Major genomic elements in *Clostridioides difficile* strain cd231108_barcode08. Circular representation of the *C. difficile* cd231108_barcode08 chromosome. The four innermost circles represent chromosome coordinates relative to cd231108_barcode08, genome coverage of mapped Nanopore reads (graph maximum value 350×), GC content, and GC skew. The degree of coloured shading indicates nucleotide identity between cd231108_barcode08 and other *C. difficile* ST2 chromosomes (Rings 5 to 8, blue), and the ST54 chromosome for strain 630 (GenBank: AM180355, purple). The outermost ring describes the positions of Cd*ISt*1 genomic elements (black), conjugative transposons (navy), genomic islands (orange), prophages and mobile elements (purple), RNA loci (grey), and regions of interest (red). Image created using BRIG[40].

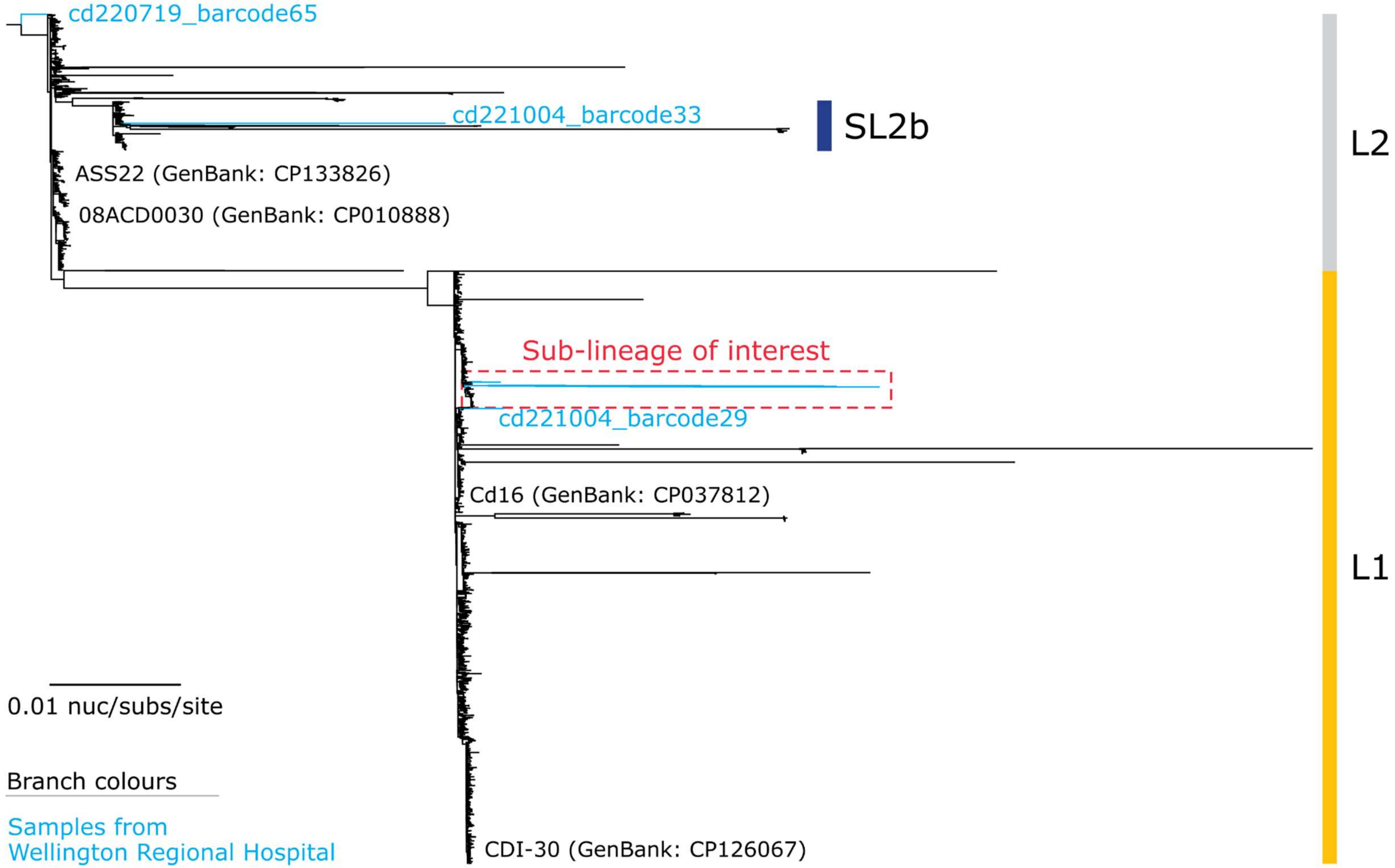

Supplementary Figure S3. Maximum likelihood phylogeny of global *Clostridioides difficile* sequence type (ST)2 genomes. The phylogeny was inferred from 14,968 core-genome single-nucleotide variants (SNVs) from 2,601 genomes. SNVs were derived from a core-genome alignment of 2,166,128 bp and are called against the chromosome of strain cd231108_barcode08 (GenBank: CP144679). Phylogeny was rooted according to the actual root by *C. difficile* strain cd220719_barcode65 (SRA: SRR27352631).

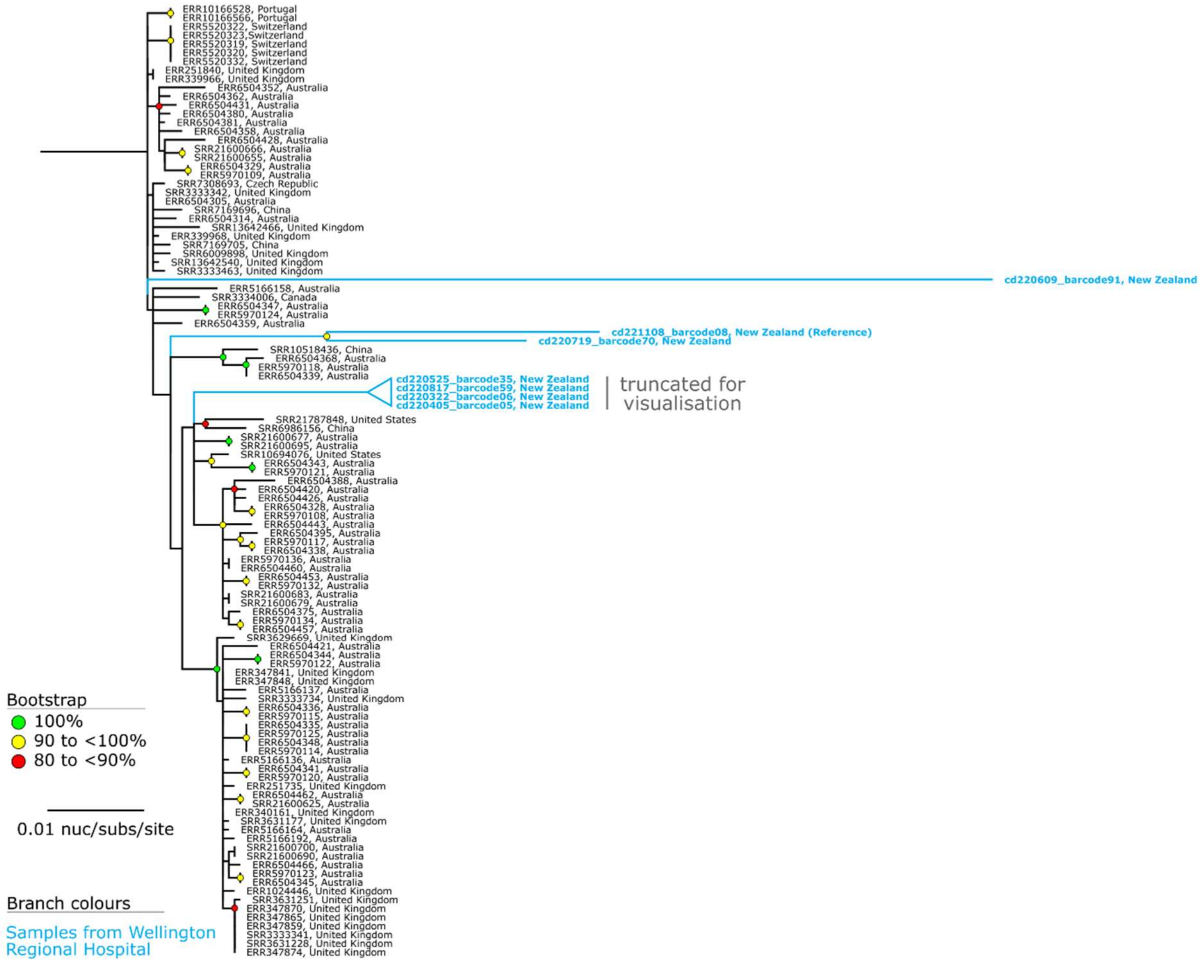

Supplementary Figure S4. Maximum likelihood phylogeny of *Clostridioides difficile* sequence type (ST)2 cases identified in Wellington Regional Hospital and placed into global context. The phylogeny was inferred from 1,675 core-genome single-nucleotide variants (SNVs) from 109 genomes. SNVs are called against the chromosome of strain cd221108_barcode08 (GenBank: CP144679). Phylogeny was rooted according to the actual root by *C. difficile* strain cd220525_barcode40 (SRA: SRR27352597), which has been truncated for visualisation. Bootstrap values >80% (1,000 replicates) are shown.

References

1. De Coster W, D'Hert S, Schultz DT, Cruts M, Van Broeckhoven C. NanoPack: visualizing and processing long-read sequencing data. *Bioinformatics* 2018;34:2666-2669. doi: 10.1093/bioinformatics/bty149.

2. Wood DE, Salzberg SL. Kraken: ultrafast metagenomic sequence classification using exact alignments. *Genome Biology* 2014;15:R46. doi: 10.1186/gb-2014-15-3-r46.

3. Sayers EW, Barrett T, Benson DA, *et al*. Database resources of the National Center for Biotechnology Information. *Nucleic Acids Research* 2009;37:D5-D15. doi: 10.1093/nar/gkn741.

4. Lin Y, Yuan J, Kolmogorov M, Shen MW, Chaisson M, Pevzner PA . Assembly of long error-prone reads using de Bruijn graphs. *Proceedings of the National Academy of Sciences* 2016;113:E8396-E8405. doi: 10.1073/pnas.1604560113.

5. Kolmogorov M, Yuan J, Lin Y, Pevzner PA. Assembly of long, error-prone reads using repeat graphs. *Nature Biotechnology* 2019;37:540-546. doi: 10.1038/s41587-019-0072-8.

6. Li H. Minimap2: pairwise alignment for nucleotide sequences. *Bioinformatics* 2018;34:3094-3100. doi: 10.1093/bioinformatics/bty191.

7. Li H. New strategies to improve minimap2 alignment accuracy. *Bioinformatics* 2021;37:4572-4574. doi: 10.1093/bioinformatics/btab705.

8. Vaser R, Sovic I, Nagarajan N, Sikic M. Fast and accurate de novo genome assembly from long uncorrected reads. *Genome Research* 2017;27:737-746. doi: 10.1101/gr.214270.116.

9. Gurevich A, Saveliev V, Vyahhi N, Tesler G. QUAST: quality assessment tool for genome assemblies. *Bioinformatics* 2013;29:1072-1075. doi: 10.1093/bioinformatics/btt086.

10. Larsen MV, Cosentino S, Rasmussen S. *et al*. Multilocus sequence typing of total-genome-sequenced bacteria. *Journal of Clinical Microbiology* 2012;50:1355-1361. doi: 10.1128/JCM.06094-11.

11. Wirth T, Falush D, Lan R. *et al*. Sex and virulence in *Escherichia coli*: an evolutionary perspective. *Molecular Microbiology* 2006;60:1136-1151. doi: 10.1111/j.1365-2958.2006.05172.x.

12. Liu B, Zheng D, Zhou S, Chen L, Yang J. VFDB 2022: a general classification scheme for bacterial virulence factors. *Nucleic Acids Research* 2022;50:D912-D917. doi: 10.1093/nar/gkab1107.

13. Gupta SK, Padmanabhan BR, Diene SM, *et al*. ARG-ANNOT, a new bioinformatic tool to discover antibiotic resistance genes in bacterial genomes. *Antimicrobial Agents and Chemotherapy* 2014;58: 212-220. doi: 10.1128/AAC.01310-13.

14. Hua X, Liang Q, Deng M. *et al*. BacAnt: A combination annotation server for bacterial DNA sequences to identify antibiotic resistance genes, integrons, and transposable elements. *Frontiers in Microbiology* 2021;12:649969. doi: 10.3389/fmicb.2021.649969.

15. Seemann T. Prokka: rapid prokaryotic genome annotation. *Bioinformatics* 2014;30:2068-2069. doi: 10.1093/bioinformatics/btu153.

16. Zhou Y, Liang Y, Lynch KH, Dennis JJ, Wishart DS. PHAST: a fast phage search tool. *Nucleic Acids Research* 2011;39:W347-W352. doi: 10.1093/nar/gkr485.

17. Arndt D, Grant JR, Marcu A, *et al*. PHASTER: a better, faster version of the PHAST phage search tool. *Nucleic Acids Research* 2016;44:W16-W21. doi: 10.1093/nar/gkw387.

18. Bouras G, Nepal R, Houtak G, Psaltis AJ, Wormald PJ, Vreugde S. Pharokka: a fast scalable bacteriophage annotation tool. *Bioinformatics* 2023;39. doi: 10.1093/bioinformatics/btac776.

19. Bertelli C, Laird MR, Williams KP, *et al*. IslandViewer 4: expanded prediction of genomic islands for larger-scale datasets. *Nucleic Acids Research* 2017:45;W30-W35. doi: 10.1093/nar/gkx343.

20. Varani AM, Siguier P, Gourbeyre E, Charneau V, Chandler M. ISsaga is an ensemble of web-based methods for high throughput identification and semi-automatic annotation of insertion sequences in prokaryotic genomes. *Genome Biology* 2011;12:R30. doi: 10.1186/gb-2011-12-3-r30.

21. Siguier P, Perochon J, Lestrade L, Mahillon J, Chandler M. ISfinder: the reference centre for bacterial insertion sequences. *Nucleic Acids Research* 2006;34:D32-D36. doi: 10.1093/nar/gkj014.

22. Rutherford K, Parkhill J, Crook J, *et al*. Artemis: sequence visualization and annotation. *Bioinformatics* 2000;16:944-945. doi: 10.1093/bioinformatics/16.10.944.

23. Treangen TJ, Ondov BD, Koren S, Phillippy AM. The Harvest suite for rapid core-genome alignment and visualization of thousands of intraspecific microbial genomes. *Genome Biology* 2014;15;524. doi: 10.1186/s13059-014-0524-x.

24. Stamatakis A. RAxML version 8: a tool for phylogenetic analysis and post-analysis of large phylogenies. *Bioinformatics* 2014;30:1312-1313. doi: 10.1093/bioinformatics/btu033.

25. Bolger AM, Lohse M, Usadel B. Trimmomatic: a flexible trimmer for Illumina sequence data. *Bioinformatics* 2014;30:2114-2120. doi: 10.1093/bioinformatics/btu170.

26. Song L, Florea L, Langmead B. Lighter: fast and memory-efficient sequencing error correction without counting. *Genome Biology* 2014;15:509. doi: 10.1186/s13059-014-0509-9.

27. Magoc T, Salzberg SL. FLASH: fast length adjustment of short reads to improve genome assemblies. *Bioinformatics* 2011;27:2957-2963. doi: 10.1093/bioinformatics/btr507.

28. Souvorov A, Agarwala R, Lipman DJ. SKESA: strategic k-mer extension for scrupulous assemblies. *Genome Biology* 2018;19:153. doi: 10.1186/s13059-018-1540-z.

29. Souvorov A, Agarwala R. SAUTE: sequence assembly using target enrichment. *BMC Bioinformatics* 2021:22:375. doi: 10.1186/s12859-021-04174-9.

30. Li H, Handsaker B, Wysoker A, *et al*. The Sequence Alignment/Map format and SAMtools. *Bioinformatics* 2009;25:2078-2079. doi: 10.1093/bioinformatics/btp352.

31. Li H, Durbin R. Fast and accurate short read alignment with Burrows-Wheeler transform. *Bioinformatics* 2009;25:1754-1760. doi: 10.1093/bioinformatics/btp324.

32. Walker BJ, Abeel T, Shea T, *et al*. Pilon: an integrated tool for comprehensive microbial variant detection and genome assembly improvement. *PLOS One* 2014;9:e112963. doi: 10.1371/journal.pone.0112963.

33. Quinlan AR, Hall IM. BEDTools: a flexible suite of utilities for comparing genomic features. *Bioinformatics* 2010;26:841-842. doi: 10.1093/bioinformatics/btq033.

34. Ye K, Schulz MH, Long Q, Apweiler R, Ning Z. Pindel: a pattern growth approach to detect break points of large deletions and medium sized insertions from paired-end short reads. *Bioinformatics* 2009;25:2865-2871. doi: 10.1093/bioinformatics/btp394.

35. Pedersen BS, Quinlan AR. Mosdepth: quick coverage calculation for genomes and exomes. *Bioinformatics* 2018;34:867-868. doi: 10.1093/bioinformatics/btx699.

36. DePristo MA, Banks E, Poplin R, *et al*. A framework for variation discovery and genotyping using next-generation DNA sequencing data. *Nature Genetics* 2011;43:491-498. doi: 10.1038/ng.806.

37. McKenna A, Hanna M, Banks E, *et al*. The Genome Analysis Toolkit: a MapReduce framework for analyzing next-generation DNA sequencing data. *Genome Research* 2010;20:1297-1303. doi: 10.1101/gr.107524.110.

38. Cingolani P, Platts A, Wang L, *et al*. A program for annotating and predicting the effects of single nucleotide polymorphisms, SnpEff: SNPs in the genome of *Drosophila melanogaster* strain w^{1118}; iso-2; iso-3. *Fly* 2012;6:80-92. doi: 10.4161/fly.19695.

39. Sarovich DS, Price EP. SPANDx: a genomics pipeline for comparative analysis of large haploid whole genome re-sequencing datasets. *BMC Research Notes* 2014;7:618. doi: 10.1186/1756-0500-7-618.

40. Alikhan NF, Petty NK, Ben Zakour NL, Beatson SA. BLAST Ring Image Generator (BRIG): simple prokaryote genome comparisons. *BMC Genomics* 2011;12:402. doi: 10.1186/1471-2164-12-402.
